# Efficacy and safety of oral metronomic etoposide in adult patients with metastatic osteosarcoma

**DOI:** 10.1002/cam4.3610

**Published:** 2020-11-25

**Authors:** Audrey Perret, Julien Dômont, Ali N. Chamseddine, Sarah N. Dumont, Benjamin Verret, Sylvain Briand, Charles Court, Thierry Lazure, Julien Adam, Carine Ngo, Caroline Even, Antonin Levy, Arnaud Bayle, Francesca Lucibello, Leila Haddag‐Miliani, Matthieu Faron, Charles Honoré, Axel Le Cesne, Olivier Mir

**Affiliations:** ^1^ Department of Cancer Medicine Gustave Roussy Cancer Institute Villejuif France; ^2^ Department of International Patients Care Gustave Roussy Cancer Institute Villejuif France; ^3^ Department of Orthopaedic Surgery Kremlin‐Bicêtre Teaching Hospital Université Paris Saclay Le Kremlin‐Bicêtre France; ^4^ Department of Pathology Kremlin‐Bicêtre Teaching Hospital Université Paris Saclay Le Kremlin‐Bicêtre France; ^5^ Department of Pathology Gustave Roussy Cancer Institute Villejuif France; ^6^ Department of Head and Neck Oncology Gustave Roussy Cancer Institute Villejuif France; ^7^ Department of Radiotherapy Gustave Roussy Cancer Institute Villejuif France; ^8^ Department of Medical Imaging Gustave Roussy Cancer Institute Villejuif France; ^9^ Department of Surgery Gustave Roussy Cancer Institute Villejuif France; ^10^ Department of Ambulatory Cancer Care Gustave Roussy Cancer Institute Villejuif France

**Keywords:** etoposide, metronomic chemotherapy, osteosarcoma, sarcoma, topoisomerase II inhibitors

## Abstract

Therapeutic options in patients with metastatic osteosarcoma are limited and effective systemic treatments are needed in this setting. The aim of this case series was to assess the efficacy and toxicity of oral metronomic etoposide in adult patients with progressive metastatic osteosarcoma. We retrospectively reviewed the electronic records of patients treated with oral metronomic etoposide (25 mg thrice daily, 3 weeks out of 4) from December 2002 to December 2018 at Gustave Roussy (Villejuif, France). The primary endpoint was progression‐free rate (PFR) at 4 months; secondary endpoints were: best response (according to RECIST v1.1), progression‐free survival (PFS), overall survival (OS) and safety. With a median follow‐up of 9.8 months, 37 patients were eligible for this analysis: 68% males, median age 42 (range: 21–75), 19% with synchronous metastases, 92% with lung metastases, median PS: 1 (range: 0–3). Median number of previous treatment lines in the metastatic setting was 1 (range: 0–4). Progression‐free rate at 4 months was 40.3% (95% CI: 24.5–56.2). Best response was partial response in 11% and stable disease in 35% of patients (disease control rate: 46%). Median PFS was 3.1 months (95% CI: 2.5–4.7) and median OS was 9.8 months (95% CI: 5.1–12.3). Toxicity profile was acceptable, with 13% grade 3 haematological toxicities (anaemia and neutropenia), without any grade 3–4 non‐haematological toxicity. In our experience, oral metronomic etoposide demonstrated effective palliation along with acceptable toxicity in patients with progressive metastatic osteosarcoma.

## Graphical abstract

Oral metronomic etoposide demonstrated effective palliation (progression‐free rate at 4 months was 40.3% (95%CI: 24.5‐56.2), along with acceptable toxicity in adult patients with metastatic osteosarcoma.

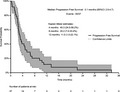

## 1 INTRODUCTION

The treatment of patients with relapsed osteosarcoma is an important clinical challenge, since no standard therapeutic strategy is established. Indeed, when metastases are not accessible to local treatments, treatment is based on palliative systemic treatments. In this setting, the prognosis remains dismal, with a progression‐free rate (PFR) at 4 months of 12% and a median overall survival (OS) of 8 months.^1^ Although phase I‐II trials conducted during the past two decades have identified potentially active agents,^2^, ^3^ the identification of new therapeutic options is still needed.

Angiogenesis plays a critical role in tumour growth and metastatic dissemination in osteosarcoma^4^, ^5^ and multi‐kinase inhibitors (MKIs) targeting the VEGF signalling pathway are under investigation in advanced osteosarcoma.^6^, ^7^, ^8^, ^9^, ^10^

Chronic, low‐dose, oral etoposide (given according to a metronomic regimen) has shown some activity against a broad spectrum of recurrent malignancies in adults (including soft‐tissue sarcoma), along with an acceptable toxicity.^11^, ^12^, ^13^ It has been suggested that metronomic administration of chemotherapy agents might exert anti‐angiogenic effects.^14^, ^15^, ^16^ Metronomic chemotherapy could also modulate the immune response by different mechanisms, encompassing the preferential depletion of regulatory T lymphocytes, increased cytotoxic activity of immune effector cells, modulation of dendritic cells and enhancement of antigen presentation.^17^, ^18^

In this context, oral metronomic etoposide might represent an interesting approach in the treatment of metastatic osteosarcoma. The objective of this retrospective, single‐centre case series was to investigate the anti‐tumour activity and safety of oral metronomic etoposide treatment in adult patients with progressive, metastatic osteosarcoma.

## 2 PATIENTS AND METHODS

We performed a retrospective study of a cohort of consecutive patients treated with oral etoposide in our institution, with the approval of the Institutional Review Board. The study was conducted in agreement to applicable laws and regulation and the European General Data Protection Regulation. Eligible patients were adults with progressive, metastatic osteosarcoma neither eligible for metastasectomy nor for a clinical trial and/or having exhausted other conventional intravenous chemotherapy options. The diagnosis of osteosarcoma was confirmed by an expert pathologist from the French Expert Pathology Group for Bone Sarcoma (NetSARC ‐ RESOS). Imaging data (CT‐scan and when applicable: MRI) were reviewed, and only patients with a confirmed progression according to RECIST 1.1^19^ in the 3 months (±2 weeks) preceding the introduction of oral metronomic etoposide were included. Demographic data, tumour characteristics (location, metastatic sites), previous treatments and outcomes (activity and safety) were recorded (data available upon request). Exclusion criteria included bone sarcoma other than high grade osteosarcoma and extra‐skeletal osteosarcoma.

### 2.1 Treatment

Patients received oral metronomic etoposide at a dose of 25 mg thrice daily, 3 weeks out of 4 (1 cycle = 28 days) or 25 mg twice daily, 2 weeks out of 3 (depending on the number of previous treatment lines; no intra‐patient dose escalation was performed). Treatment was maintained until disease progression or intolerable toxicity. The concomitant intake of treatments that might affect the pharmacokinetics of oral etoposide was collected. Notably, since the influence of gastric pH on the bioavailability of oral etoposide has not been studied, we examined the intake of gastric acid suppressants.

There was no systematic prescription of G‐CSF or anti‐emetics other than metoclopramide (at approved doses). Laboratory tests (blood cells count, serum chemistry, renal function, liver function) were performed monthly or more frequently if clinically needed. Tumour evaluation (using CT and/or MRI) was performed every 2 cycles or earlier if clinically needed.

### 2.2 Evaluation criteria

The primary endpoint was PFR at 4 months. Secondary endpoints were: objective response rate (ORR), progression‐free survival (PFS), OS and safety. Response to treatment was evaluated according to RECIST 1.1. Disease control rate (DCR) was defined as the sum of objective responses and stable diseases. PFS was defined as the time from the initiation of oral metronomic etoposide to disease progression, death or last follow‐up. OS was defined as the time from the initiation of oral metronomic etoposide to death or last follow‐up. Safety data (treatment‐related clinical and biological toxicities) were collected from medical records and retrospectively graded according to the National Cancer Institute Common Terminology Criteria for Adverse Events (NCI‐CTCAE) version 5.0.

### 2.3 Statistical analysis

Descriptive statistics were used to analyse patient characteristics: median, ranges, 95% confidence intervals (95% CI). A univariate analysis was conducted to assess the influence of the baseline parameters on PFR at 4 months and OS. Variables influencing these outcomes with a *p* < 0.20 were implemented in a multivariate analysis. Tests were performed using the NCSS 2020^TM^ software (https://www.ncss.com/). Survival data were estimated using the Kaplan–Meier method with the log‐rank test. A *p* < 0.05 was considered statistically significant.

## 3 RESULTS

### 3.1 Patient characteristics

From December 2002 to December 2018, 37 eligible patients were identified: 25 males (68%), with a median age of 42 years (range: 21–75). The baseline characteristics of patients are summarised in Table 1. In patients with metachronous metastases (*n* = 30, 81%), the median time to metastatic relapse was 21.5 months (range: 9–135). Overall, the median time between the diagnosis of metastases and the initiation of oral metronomic etoposide was 10 months (range: 0–160). Patients had received a median number of previous lines of 1 (range: 0–4) for metastatic disease. Most patients had received doxorubicin (*n* = 37, 100%), ifosfamide (*n* = 32, 86.5%) and cisplatin (*n* = 36, 97%). Twenty‐one patients (57%) had also previously received intravenous etoposide. Five patients had received one or more MKIs (sorafenib, sunitinib, regorafenib or cabozantinib).

**Table 1:** Patient's baseline characteristics

		Patients (n = 37)
Age (years): median (range)		42 (21–75)
Gender: *n* (%)	Male	25 (68%)
	Female	12 (32%)
Performance status: *n* (%)	0	8 (22%)
	1	16 (43%)
	2	7 (19%)
	3	5 (13%)
	Missing	1 (3%)
Primary site: *n* (%)	Femur	14 (38%)
	Tibia	7 (19%)
	Humerus	3 (8%)
	Maxillary/mandibular	6 (16%)
	Pelvis	5 (13%)
	Spine	2 (6%)
Number of metastatic sites: *n* (%)	1	24 (65%)
	2	11 (30%)
	≥3	2 (5%)
Metastatic sites: *n* (%)	Lung	34 (92%)
	Bones	15 (40%)
	Other	4 (11%)
Number of previous systemic treatment lines for metastatic disease: *n* (%)	0	5 (13.5%)
	1	18 (49%)
	2	9 (24%)
	≥3	5 (13.5%)
Previous systemic treatments in neoadjuvant/adjuvant and metastatic settings: *n* (%)	Doxorubicin	37 (100%)
	Ifosfamide	32 (86.5%)
	Cisplatin	36 (97%)
	IV Etoposide	21 (57%)
	HDMTX	4 (11%)
	Gemcitabine	7 (19%)
	Taxanes	2 (5%)
	Oral CPM	15 (40%)
	MKI	5 (13.5%)
	Other	11 (30%)

Abbreviations: CPM, cyclophosphamide; HDMTX, high‐dose methotrexate; IV, intravenous; MKI, multi‐kinase inhibitors targeting the VEGF receptors; UNK, unknown.

### 3.2 Treatment disposition

The median duration of treatment with oral metronomic etoposide was 3.1 months (range: 0–30). All patients but 3 (who rapidly progressed) received at least one month of treatment.

Etoposide was given according to the 25 mg regimen thrice daily, 3 weeks out of 4, in 32 patients (86.5%). In four patients, palliative radiotherapy was required during treatment with oral etoposide (for bone pain on non‐target lesions in all cases) and etoposide dose was reduced to 25 mg daily during irradiation. Of the 30 patients for whom information was available in the medical record, 8 (27%) received concomitant gastric acid suppressants. Twenty‐five patients (67%) were able to receive one or more subsequent treatment lines after progression under oral metronomic etoposide.

### 3.3 Efficacy

**Figure 1:**
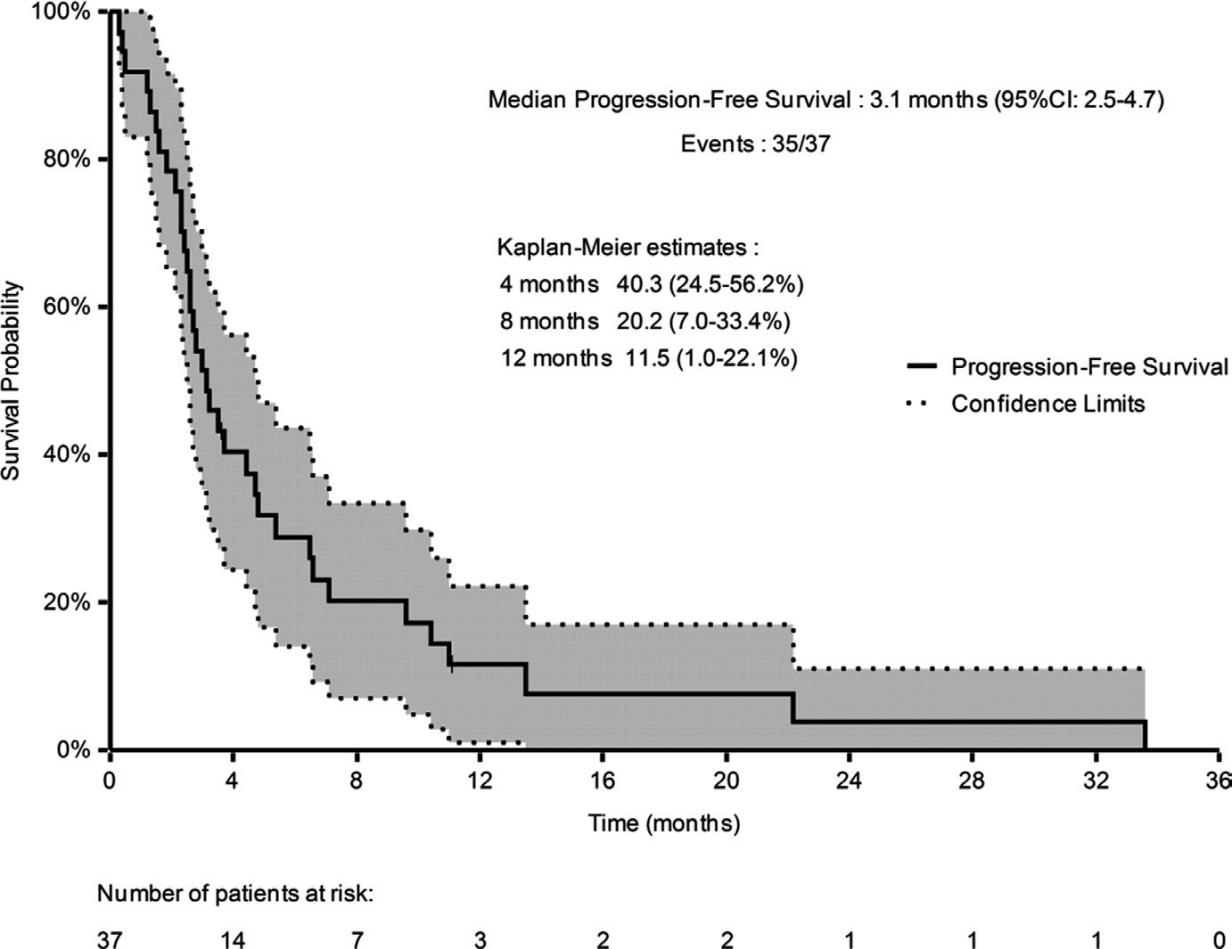
Progression‐free survival (Kaplan–Meier estimate), *n* = 37

**Figure 2:**
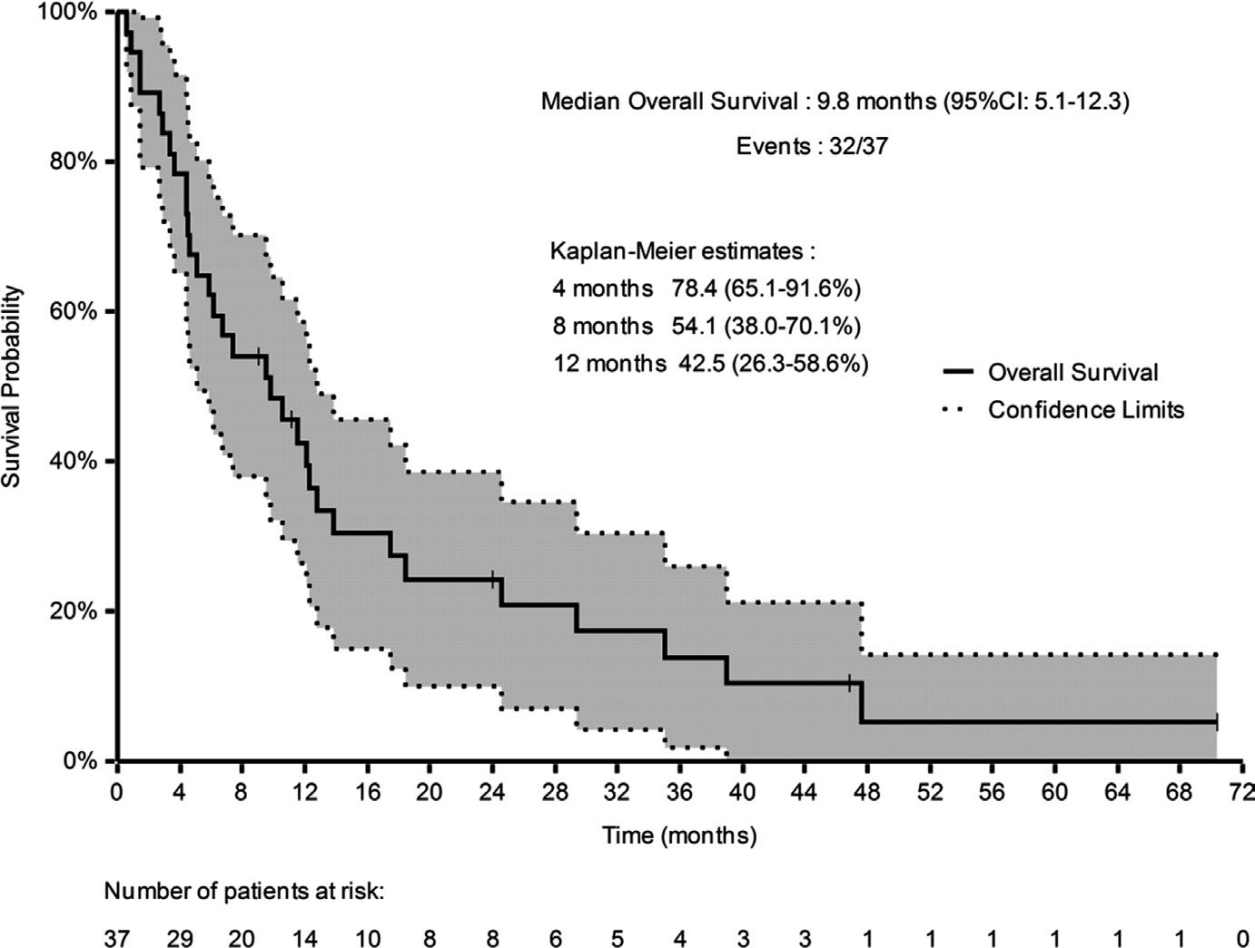
Overall survival (Kaplan–Meier estimate), *n* = 37

The median follow‐up was 9.8 months (range: 1–71). PFR at 4 months was 40.3% (95% CI: 24.5–56.2). Best response was partial response in 11% and stable disease in 35% of patients (DCR: 46%). Median PFS was 3.1 months (95 CI%: 2.5–4.7, Figure 1). Median OS was 9.8 months (95% CI: 5.1–12.3, Figure 2).

Of 21 patients previously treated with intravenous etoposide, three (14%) had a partial response and another four (19%) had stable disease as best response. Of five patients previously treated with MKIs targeting the VEGF receptors, one experienced a stable disease for 6 months under oral metronomic etoposide; the remaining four had progressive disease. Conversely, of four patients progressing under oral metronomic etoposide subsequently treated with MKIs, two experienced partial responses and another two stable disease.

The number of previous treatment lines (*p* = 0.02) and baseline performance status (*p* < 0.01) were significantly associated to PFR at 4 months by univariate analysis. By multivariate analysis, no variable significantly influenced PFR at 4 months.

### 3.4 Toxicity

Safety data are shown in Table 2. Seventeen patients (46%) had clinical and/or haematological toxicity. Grade 3 toxicities were exclusively haematological, with three patients (8%) having grade 3 anaemia and two patients (5%) having grade 3 neutropenia (including one febrile neutropenia). One patient stopped treatment due to prolonged grade 3 thrombocytopenia. No grade 4–5 toxicity was observed.

**Table 2:** Adverse events related to oral metronomic etoposide

	Grade 1–2	Grade 3	Total
Clinical toxicity: *n*			15 (40.5%)
Fatigue	7 (19%)	0	7 (19%)
Anorexia	2 (5%)	0	2 (5%)
Nausea‐Vomiting	2 (5%)	0	2 (5%)
Mucositis	1 (3%)	0	1 (3%)
Alopecia	3 (8%)	0	3 (8%)
Haematological toxicity: *n*			16 (43%)
Neutropenia	4 (11%)	2^a^ (5%)	2 (5%)
Anaemia	7 (19%)	3 (8%)	10 (27%)
Thrombocytopenia	4 (11%)	0	4 (11%)

^a^ Including one febrile neutropenia.

Finally, there was no impact of gastric acid suppressants intake on toxicity or anti‐tumour activity.

## 4 DISCUSSION

In this retrospective study of 37 patients with metastatic osteosarcoma, we observed evidence of anti‐tumour activity of oral metronomic etoposide in adult patients with metastatic osteosarcoma, with an acceptable toxicity profile.

The primary objective of this study was to evaluate the anti‐tumour efficacy of metronomic oral etoposide by determining the PFR at 4 months, which was 40.3% (95% CI: 24.5–56.2). We have chosen this primary endpoint because, since 2008, the osteosarcoma community of experts has favoured the use of PFS or PFR as an endpoint in Phase II trials in recurrent osteosarcoma.^2^, ^20^ Previous studies also suggest that the median PFS of an inactive drug is about 6 weeks in metastatic osteosarcoma,^21^, ^22^ and that an active drug should double this median (i.e., 12 weeks or 3 months) to be considered worth being further investigated. The median PFS in the present series was 3.1 months, supporting the efficacy of oral metronomic etoposide. However, the present retrospective analysis is not a phase 2 trial, and these data should be interpreted with caution.

Indeed, and recently, other oral anti‐cancer agents have also met these endpoints in recurrent osteosarcoma, as summarised in Table 3. In particular, MKIs targeting the VEGF receptors^7^, ^8^, ^10^ are considered promising in this setting. However, their activity has to be balanced with their cost and their toxicity (for instance, 64% of patients experience grade 3 or higher toxicity with regorafenib^7^). With regards to the toxicity profile of other chemotherapy schedules used in relapsed osteosarcoma, the gemcitabine‐docetaxel and the gemcitabine‐sirolimus doublets resulted in 25% of grade 4 haematological toxicities and >20% of grade ≥3 toxicities, respectively.^20^, ^23^ In the present series, the toxicity of oral metronomic etoposide was acceptable, with 13% of patients experiencing grade ≥3 toxicities (haematological) and no grade 4–5 toxicities.

**Table 3:** Summary of recent studies in metastatic osteosarcoma

Study	Study design	Drug	Number of patients	ORR: n (%)	Median PFS	PFR at 4 months	Median OS
Davis et al.^9^	Phase II, randomised, placebo‐controlled	Regorafenib	22	2 (9%)	3.6 months 95% CI: 2–7.6	44.4%	11.1 months 95% CI: 4.7–26.7
Duffaud et al.^8^	Phase II, randomised, placebo‐controlled	Regorafenib	26	2 (8%)	16.4 weeks 95% CI: 8–27.3	NR	11.3 months 95% CI: 5.9–23.9
Grignani et al.^6^	Phase II, single arm	Sorafenib +Everolimus	38	2 (5%)	5 months 95% CI: 2–7	NR	11 months 95% CI: 8–15
Grignani et al.^7^	Phase II, single arm	Sorafenib	35	3 (8%)	4 months 95% CI: 2–5	46%	7 months 95% CI: 7–8
Italiano et al.^10^	Phase II, single arm	Cabozantinib	42	5 (12%)	6.2 months 95% CI: 5.4–8.2	33%	10.6 months 95% CI: 7.2–13.2
Longhi et al.^26^	Retrospective	Pazopanib	15	1 (7%)	6 months (range=2–10)	NR	7 months (range =2–15)
Xie et al.^27^	Phase II, single arm	Apatinib	37	16 (43%)	4.5 months 95% CI: 3.5–6.3	57%	9.9 months 95% CI: 7.9–18.9
Martin‐Broto et al.^20^	Phase II, single arm	Gemcitabine + Sirolimus	35	2 (6%)	2.3 months 95% CI: 0–5.2	44%	7.1 months 95% CI: 2.8–11.4
Penel‐Page et al.^28^	Retrospective	Sirolimus ± oral CPM	23 (5 received sirolimus alone)	3 (13%)	3 months 95% CI: 2–5.4	NR	NR

Abbreviations: CPM, cyclophosphamide; NR, not reported; objective response rate; ORR; OS, overall survival; PFR, progression‐free rate; PFS, progression‐free survival.

Moreover, its cost is also lower than that of MKIs; as an illustration, the monthly cost of oral etoposide (according to the schedule described above) in France is 356 euros.^24^

Interestingly, we observed no evidence of cross‐resistance between MKIs and oral metronomic etoposide, despite its putative anti‐angiogenic activity. Likewise, there was no evidence of cross‐resistance with IV etoposide.

In this context, we believe that MKIs have potential for further trial exploration (especially in earlier disease settings), whereas oral etoposide will likely remain a palliative (safe and tolerable) regimen. Clinical trials exploring the use of metronomic etoposide as maintenance treatment in relapsed osteosarcoma have been performed in small samples^25^ and should probably be replicated in larger cohorts. Besides, regorafenib is also evaluated as a maintenance therapy in bone sarcoma in an ongoing randomised phase 2 trial (NCT04055220).

Finally, these results and their interpretation are limited by their retrospective nature and the small number of patients. There is also heterogeneity of patients in the present series, as well as in previous studies, particularly in terms of primary tumour sites, time to metastatic disease and sensitivity to first‐line treatment. This heterogeneity represents a potential bias in the interpretation of the results and requires further clinical investigations.

### 4.1 Implications for clinical care

Oral metronomic etoposide is an attractive option in terms of schedule of administration (requiring no hospitalisation) and might represent an additional therapeutic option in relapsed osteosarcoma adult patients.

## 5 CONCLUSION

In our experience, oral metronomic etoposide demonstrated effective palliation along with acceptable toxicity in patients with progressive metastatic osteosarcoma. It might be considered as an additional therapeutic option in this population.
